# *DNMT3A* Co‐Mutation and Real‐World Clinical Outcomes With Gilteritinib in FLT3‐Mutated Acute Myeloid Leukaemia Across Molecular Subgroups

**DOI:** 10.1002/jha2.70374

**Published:** 2026-08-05

**Authors:** Francesca Duca, Beatrice Manghisi, Andrea Palasciano, Anna Mochi, Matteo Parma, Rosa Greco, Roberto Cairoli, Carlo Gambacorti‐Passerini, Monica Fumagalli

**Affiliations:** ^1^ Department of Medicine and Surgery University of Milano‐Bicocca Monza Italy; ^2^ Haematology and Transplantation Unit Fondazione IRCCS San Gerardo dei Tintori Monza Italy; ^3^ Division of Clinical and Translational Research Department of Medicine McGill University Montreal Canada; ^4^ Lady Davis Institute for Medical Research and Segal Cancer Center Jewish General Hospital Montreal Canada; ^5^ Department of Haematology ASST Grande Ospedale Metropolitano Niguarda Niguarda Cancer Centre Milano Italy

**Keywords:** acute myeloid leukaemia, *DNMT3A*, FLT3 inhibitor, gilteritinib, molecular subgroups, real‐world

## Abstract

**Introduction:**

Molecular co‐mutations modulate outcomes in *FLT3*‐mutated AML treated with gilteritinib; real‐world data stratified by *DNMT3A* co‐mutation status are lacking.

**Methods:**

We retrospectively analysed 29 adults with R/R *FLT3*‐mutated AML treated at two Italian centres. Sixteen patients with complete molecular profiling were stratified into three subgroups by *DNMT3A* and *NPM1* co‐mutation status.

**Results:**

Overall response rate was 89.7% (26/29). *DNMT3A*‐mutated subgroups showed prolonged OS from gilteritinib initiation versus wild‐type group (median not reached vs. 8.8 months; 12‐month OS 78% vs. 17%).

**Conclusions:**

These hypothesis‐generating data suggest that *DNMT3A* co‐mutation status is associated with differential gilteritinib outcomes, supporting comprehensive molecular profiling in *FLT3*‐mutated AML.

**Trial Registration:**

The authors have confirmed clinical trial registration is not needed for this submission

## 1 Introduction

Comprehensive genomic profiling has identified recurrent co‐mutation patterns in acute myeloid leukaemia (AML) with distinct prognostic implications. Papaemmanuil et al. demonstrated that the *FLT3‐ITD/NPM1/DNMT3A* triple‐mutant genotype, observed in approximately 6% of AML cases, was associated with significantly inferior outcomes compared with other *FLT3*‐positive subgroups, with median overall survival (OS) of approximately 12 months from diagnosis despite intensive chemotherapy [1]. Subsequent cohorts confirmed this adverse prognostic impact in the pre‐targeted therapy era [2, 3, 4].

Garg et al. showed that *FLT3‐*ITD*, NPM1* and *DNMT3A* mutations synergistically upregulate hepatic leukaemia factor (HLF), a key regulator of leukaemic stem cell self‐renewal and proliferation [5]. Transcriptomic profiling has further revealed enrichment for *HOX* family genes and histone *H1* signatures in triple‐mutant cases, supporting a model where epigenetic dysregulation (*DNMT3A*), altered transcriptional programmes (*NPM1*) and constitutive proliferative signalling (*FLT3‐*ITD) cooperate to drive leukaemogenesis and confer vulnerability to FLT3 inhibition [6].

Of note, Othman et al. analysed 1357 patients with *NPM1*‐mutated AML and confirmed that the triple‐mutant genotype (approximately 23% of those sequenced) was associated with poor outcomes, with a 3‐year OS of 52%. *DNMT3A* mutations predicted relapse even in MRD‐negative patients, supporting *DNMT3A*‐based molecular stratification [7].

The selective FLT3 inhibitor gilteritinib has substantially improved outcomes in relapsed/refractory (R/R) *FLT3*‐mutated AML. In the pivotal ADMIRAL trial, gilteritinib demonstrated superior OS compared with salvage chemotherapy (median OS 9.3 vs. 5.6 months), with successful bridging to allogeneic transplantation in 25.5% of patients [8]. Post hoc molecular analyses revealed that patients harbouring concurrent *DNMT3A* and *NPM1* mutations achieved particularly favourable outcomes with gilteritinib [8, 9].

Real‐world data from Cairoli et al. (205 Italian patients) showed a median OS of 10.3 months and transplantation in 48% of bridge‐intent patients, while *TP53* co‐mutations retained adverse prognostic impact [10]. Recent real‐world cohorts have suggested that triple‐mutant cases may derive exceptional benefit from *FLT3* inhibition [11]. However, triple‐mutant outcomes remain poorly characterised, and current risk models inadequately predict response to *FLT3* inhibitors based on co‐mutation status alone [12].

## 2 Methods

We retrospectively analysed 29 adult patients with R/R *FLT3*‐mutated AML treated with gilteritinib 120 mg daily at two Italian centres, Fondazione IRCCS San Gerardo dei Tintori (*n* = 14) and ASST Grande Ospedale Metropolitano Niguarda (*n* = 15) between January 2020 and December 2025.

*FLT3‐*ITD*/*TKD mutations were confirmed by PCR‐based fragment analysis and, when available, a 54‐gene NGS myeloid panel at diagnosis; *FLT3* mutational status was reassessed at relapse. Patients with *FLT3‐*ITD and complete molecular profiling (*n* = 16) were classified into three molecular subgroups: Group 1 (*FLT3‐*ITD/*NPM1*/*DNMT3A* triple‐mutant, *n* = 5), Group 2 (*FLT3‐*ITD/*DNMT3A* with *NPM1* wild‐type, *n* = 4), and Group 3 (*FLT3‐*ITD without *DNMT3A* mutations, *n* = 7; 3 *NPM1*‐mutated and 4 *NPM1* wild‐type).

Patients received gilteritinib as bridge‐to‐transplant (*n* = 10, 34.5%), for post‐transplant relapse (*n* = 8, 27.6%), or as definitive therapy in transplant‐ineligible patients (*n* = 11, 37.9%).

We employed a dual‐endpoint approach: the primary endpoint was OS from gilteritinib initiation (OS‐gilt), while OS from diagnosis (OS‐diag) served as a secondary endpoint for comparison with pre‐targeted‐therapy cohorts. OS‐gilt was defined from treatment initiation to death, and PFS from treatment initiation to relapse, progression or death. Response criteria followed European LeukemiaNet (ELN) 2022 recommendations [13] and survival was estimated by Kaplan–Meier; survivors were censored at last follow‐up. An exploratory log‐rank test compared OS‐gilt between *DNMT3A*‐mutated (Groups 1 + 2, *n* = 9) and *DNMT3A* wild‐type patients (Group 3, *n* = 7). This retrospective study was conducted in accordance with the Declaration of Helsinki. Individual consent was waived given the retrospective, anonymised study design.

## 3 Results

Baseline characteristics are summarised in Table 1. Median age was 66 years (range 28–81); 65.5% were female. All patients harboured *FLT3* mutations: 24 (82.8%) *FLT3‐*ITD, 3 (10.3%) *FLT3‐*TKD and 2 (6.9%) concurrent *FLT3‐*ITD and TKD. No *TP53* co‐mutations were identified among NGS‐profiled patients. Most patients (69.0%) received intensive induction chemotherapy, while 27.6% received hypomethylating agents plus venetoclax. Twenty‐seven of the 29 treated patients were evaluable for response, as two died before response assessment. Among evaluable patients, the ORR was 96.3% (95% CI, 81.7%–99.3%), with CR in 22 patients (81.5%), CRi in 1 (3.7%), PR in 3 (11.1%) and one non‐responder. In the intention‐to‐treat population (*n* = 29), the ORR was 89.7% (26/29). With median follow‐up of 15.1 months (range 1.3–49.7), median OS‐gilt was 9.4 months, and median progression‐free survival (PFS) was 9.4 months. Median OS‐diag was 19.1 months (range, 4.7–58.3). At data cut off, 10 patients remained alive.

**TABLE 1:** Baseline characteristics of 29 adult patients with relapsed/refractory *FLT3*‐mutated AML treated with gilteritinib, stratified by molecular subgroup (*n* = 16 classifiable).

Characteristic	Overall cohort (*N* = 29)	Group 1 (Triple‐mut) (*n* = 5)	Group 2 (*DNMT3A*+) (*n* = 4)	Group 3 (*DNMT3A*− wt) (*n* = 7)
Age at diagnosis, median (range), years	66 (28–81)	69 (44–71)	56 (46–71)	57 (48–75)
Sex, female, *n* (%)	19 (65.5)	3 (60.0)	2 (50.0)	5 (71.4)
**Diagnosis, *n* (%)**				
AML de novo	21 (72.4)	5 (100)	2 (50.0)	4 (57.1)
AML‐MR	8 (27.6)	0	2 (50.0)	3 (42.9)
**Karyotype, *n* (%)**				
Normal	19 (65.5)	5 (100)	1 (25.0)	4 (57.1)
Complex	3 (10.3)	0	1 (25.0)	2 (28.6)
Other/abnormal	7 (24.1)	0	2 (50.0)	1 (14.3)
***FLT3* mutation, *n* (%)**				
*FLT3*‐ITD	24 (82.8)	5 (100)	4 (100)	5 (71.4)
*FLT3*‐TKD	3 (10.3)	—	—	—
Concurrent ITD/TKD	2 (6.9)	—	—	2 (28.6)
**Prior therapy, *n* (%)**				
Intensive induction (7 + 3)	20 (69.0)	4 (80.0)	3 (75.0)	5 (71.4)
Non‐intensive (HMA + VEN)	8 (27.6)	1 (20.0)	0	2 (28.6)
Other	1 (3.4)	0	1 (25.0)	0
Prior allo‐HSCT	10 (34.5)	0	1 (25)	5 (71.4)
Prior FLT3i exposure	19 (65.5)	3 (60.0)	4 (100.0)	6 (85.7)
**Gilteritinib setting, *n* (%)**				
Bridge‐to‐transplant	10 (34.5)	3 (60.0)	3 (75.0)	3 (42.9)
Post‐transplant relapse	8 (27.6)	0	1 (25.0)	3 (42.9)
Unfit for transplant	11 (37.9)	2 (40.0)	0	1 (14.3)

Abbreviations: AML, acute myeloid leukaemia; AML‐MR, AML with myelodysplasia‐related changes; DNMT3A, DNA methyltransferase 3 alpha; FLT3, FMS‐like tyrosine kinase 3; FLT3i, FLT3 inhibitor; HMA, hypomethylating agents; HSCT, haematopoietic stem cell transplantation; ITD, internal tandem duplication; NPM1, nucleophosmin 1; TKD, tyrosine kinase domain; VEN, venetoclax.

**FIGURE 1:**
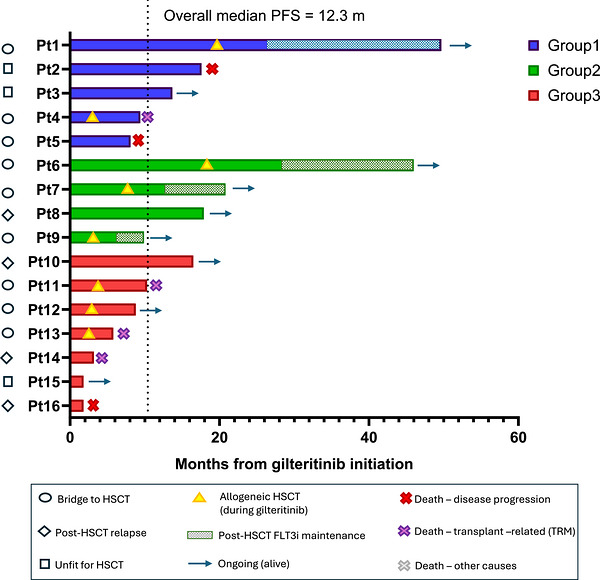
Swimmer plot of individual patient outcomes on gilteritinib, stratified by molecular subgroup (*n* = 16).

Among the 16 patients with *FLT3‐*ITD and complete molecular profiling, *FLT3* mutational status was confirmed at relapse: 14 retained *FLT3*‐ITD, whereas two patients with concurrent *FLT3*‐ITD/TKD at diagnosis lost *FLT3*‐ITD while retaining *FLT3*‐TKD at relapse. Median OS‐gilt was 17.6 months (range, 1.8–49.7), median PFS was 12.3 months, and median OS‐diag was 21.4 months (range, 6.7–58.3). Survival differed across molecular subgroups (Figure 1).

Group 1 patients (*FLT3‐*ITD*/NPM1/DNMT3A*, *n* = 5) achieved a 100% response rate with median OS‐gilt of 17.6 months (range, 8.1–49.7), median PFS of 12.3 months, and median OS‐diag of 19.1 months (range, 11.0–58.3). Despite three deaths, the longest survivor belonged to this group (ongoing at 49.7 months). OS‐gilt exceeded the 15.1‐month gilteritinib benchmark for *DNMT3A*/*NPM1* co‐mutated patients in ADMIRAL [9], despite this genotype's historically poor prognosis from diagnosis in the pre‐targeted‐therapy era (∼12 months [1]).

Group 2 patients (*FLT3‐*ITD*/DNMT3A* with *NPM1* wild‐type, *n* = 4) demonstrated numerically superior outcomes with all four patients alive at data cut off; medians were not reached (follow‐up 9.9–46.0 months from gilteritinib, 12.3–53.0 months from diagnosis). These findings suggest that *NPM1* status may modulate response within *DNMT3A*‐mutated AML, although baseline characteristics and prior treatment may also have contributed.

In Group 3 (*FLT3*‐ITD without *DNMT3A* mutations, *n* = 7), all six evaluable patients achieved a response (CR in 4, PR in 2; ORR 85.7%, 6/7; 1 was not evaluable because of early death). Despite high response rates, survival outcomes were inferior, with median OS‐gilt of 8.8 months (range, 1.8–16.5), median PFS of 7.7 months, and median OS‐diag of 21.3 months (range, 6.7–26.2). Of the five deaths, three were transplant‐related.

On an exploratory log‐rank analysis, *DNMT3A*‐mutated patients (Groups 1 + 2, *n* = 9) demonstrated prolonged OS‐gilt compared with *DNMT3A* wild‐type patients (Group 3, *n* = 7; median not reached vs. 8.8 months; 12‐month OS 78% vs. 17%; *p* = 0.014) (Figure S1), demonstrating that *DNMT3A* co‐mutation is associated with favourable outcomes in this cohort, thereby warranting validation in larger cohorts.

Among *NPM1*‐mutated patients with available MRD data by RT‐qPCR, one of five Group 1 patients achieved MRD‐negative CR, two MRD‐positive CR, and two CR without MRD assessment. Among the two evaluable *NPM1*‐mutated Group 3 patients, one achieved MRD‐positive CR and one partial response; no patient achieved MRD negativity.

Considering the overall cohort, among bridge‐to‐transplant patients (*n* = 10), nine (90%) successfully proceeded to allogeneic transplantation, compared with historical benchmarks [8, 10, 14]. Five transplanted patients received post‐transplant FLT3 inhibitor maintenance: four resumed gilteritinib (median 3.5 months after transplant, range 0.9–4.0) and one received sorafenib (18 days after transplant) following gilteritinib‐associated myocarditis. All remain alive and in continuous remission at last follow‐up.

Of 19 deaths, six were due to disease progression, five to transplant‐related complications, and eight to other causes, predominantly infections. Among the five transplant‐related deaths, three patients had achieved complete remission prior to fatal events. No deaths were treatment‐related. Gilteritinib was well tolerated; Grade 3–4 haematological and infectious toxicities (CTCAE v5.0) were common, with dose modifications in approximately 25% of patients. No differentiation syndrome was observed.

## 4 Discussion

These findings support the previously described HLF‐mediated biological model [5], suggesting that FLT3 inhibition may preferentially target this oncogenic dependency despite poor outcomes with conventional chemotherapy. Recent real‐world analyses similarly reported higher remission rates and superior OS in triple‐mutant patients treated with FLT3 inhibitors [11]. These data suggest *DNMT3A* co‐mutation as a potential predictor of gilteritinib response. However, the numerically superior outcomes in Group 2 compared with Group 1 should be interpreted cautiously given the small sample size, and the role of *NPM1*co‐mutation requires further validation in larger cohorts.

Limitations include the small sample size, retrospective design, and heterogeneity in prior treatments. The small subgroup size (*n* = 16) limits statistical power; hence, the findings remain hypothesis‐generating.

Furthermore, OS from diagnosis is subject to selection bias, as patients dying before gilteritinib were excluded by definition; this endpoint was included solely to allow contextual comparison with pre‐targeted‐therapy era cohorts.

Group 3 was molecularly and cytogenetically heterogeneous, including both *NPM1‐*mutated (*n* = 3) and *NPM1*‐wild‐type (*n* = 4) patients, as well as two patients with complex karyotype; these baseline differences may have contributed to the inferior outcomes, and further stratification was precluded by sample size.

Complete molecular profiling was not available for all patients, reflecting real‐world limitations in NGS availability and restricting subgroup analyses.

Finally, systematic *FLT3‐*ITD MRD monitoring by NGS was unavailable; MRD assessment was limited to *NPM1*‐mutated patients by RT‐qPCR, consistent with institutional practice. This is a noted limitation given MORPHO trial evidence that peri‐transplant *FLT3‐*ITD MRD positivity predicted relapse risk and benefit from gilteritinib maintenance [15].

## 5 Conclusion

Our bicentric real‐world study suggests that *DNMT3A* co‐mutation is associated with favourable gilteritinib response in *FLT3‐*ITD AML, warranting prospective validation as a predictive biomarker. The historically poor‐risk *FLT3‐*ITD*/NPM1/DNMT3A* genotype showed high response rates with gilteritinib, as did *DNMT3A*‐mutated, *NPM1*‐wild‐type patients, possibly reflecting FLT3 dependency. All five patients receiving post‐transplant FLT3 inhibitor maintenance remain in continuous remission, supporting maintenance strategies in high‐risk molecular subsets. These findings support comprehensive molecular profiling and risk‐stratified approaches in FLT3‐mutated AML.

## Author Contributions

F.D. and M.F. designed the study. F.D. collected and analysed the data and drafted the manuscript. A.M. contributed to data collection. M.F., M.P., B.M., A.P., R.G. and R.C. provided clinical expertise. C.G.‐P. supervised the study. All authors critically revised the manuscript and approved the final version.

## Funding

The authors have nothing to report.

## Ethics Statement

This study was conducted in accordance with the Declaration of Helsinki.

## Consent

Individual patient consent was waived given the retrospective and anonymised nature of this study.

## Conflicts of Interest

Roberto Cairoli reports consulting fees from AbbVie, Gilead, Pierre Fabre, Gentili Pharma, Celgene, and Menarini Stemline; and fees for professional activities from Beigene, Daiichi Sankyo, and Servier. The other authors declare no conflicts of interest.

## Supporting information




**Supporting File**: jha270374‐sup‐0001‐figureS1.pdf.

## Data Availability

Data are available upon request to the corresponding author.
