# Supplementary material for: DNMT3A Co‐Mutation and Real‐World Clinical Outcomes With Gilteritinib in FLT3‐Mutated Acute Myeloid Leukaemia Across Molecular Subgroups
Source: EJHaem. 2026 Aug 5;7(4):e70374. doi: 10.1002/jha2.70374 (PMC13439513; doi:10.1002/jha2.70374)
Supplement: Supplementary file 1 [file JHA2-7-e70374-s001.pdf]

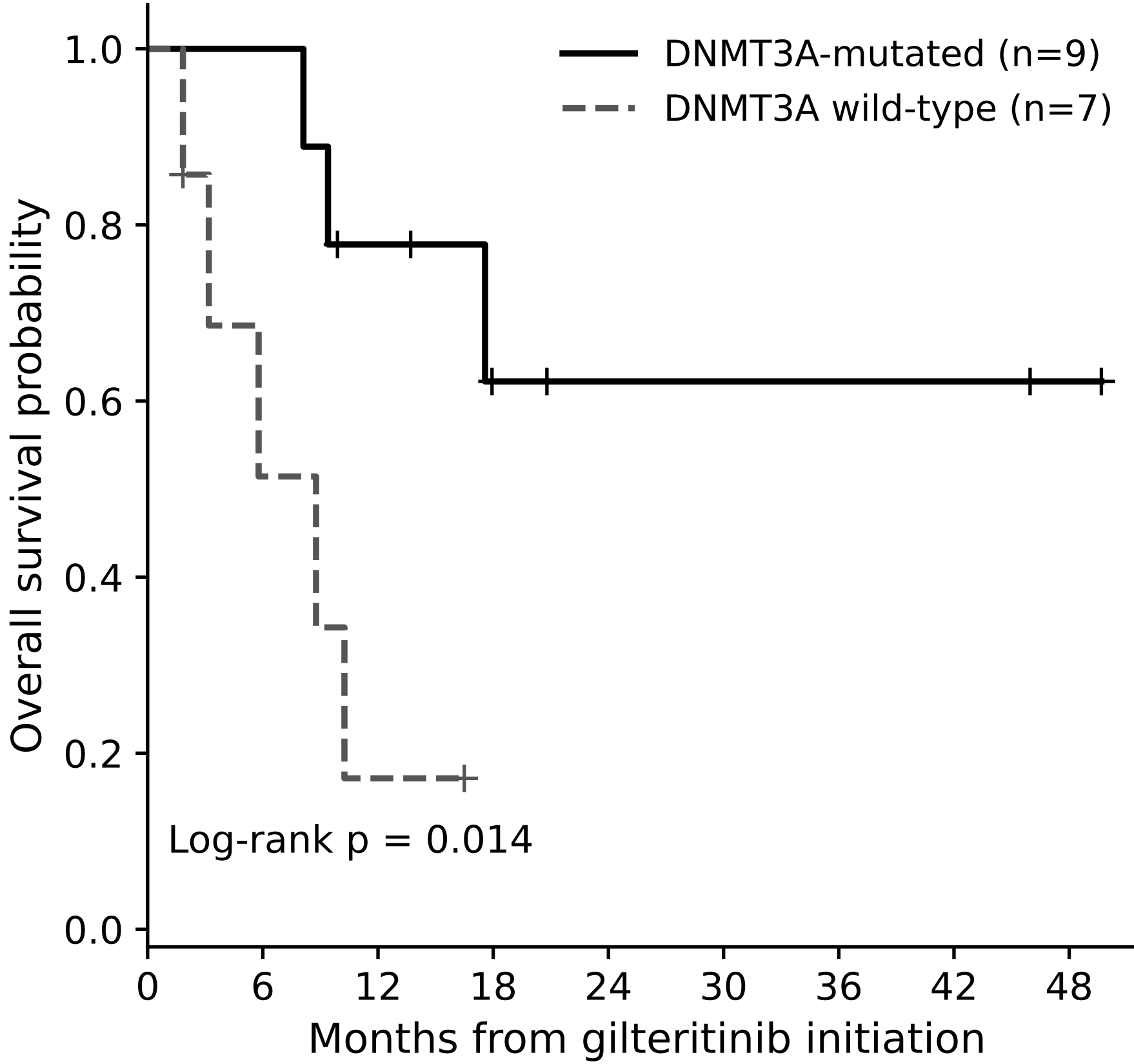


| At risk | | | | | | | | | |
|---|---|---|---|---|---|---|---|---|---|
| DNMT3A-mutated (n=9) | 9 | 9 | 6 | 3 | 2 | 2 | 2 | 2 | 1 |
| DNMT3A wild-type (n=7) | 7 | 3 | 1 | 0 | 0 | 0 | 0 | 0 | 0 |
